# Regioselective and water-assisted surface esterification of never-dried cellulose: nanofibers with adjustable surface energy†

**DOI:** 10.1039/d1gc02292j

**Published:** 2021-08-02

**Authors:** Marco Beaumont, Caio G. Otoni, Bruno D. Mattos, Tetyana V. Koso, Roozbeh Abidnejad, Bin Zhao, Anett Kondor, Alistair W. T. King, Orlando J. Rojas

**Affiliations:** Department of Chemistry, Institute of Chemistry for Renewable Resources, University of Natural Resources and Life Sciences Vienna (BOKU) Konrad-Lorenz-Straße 24 A-3430 Tulln Austria; Department of Bioproducts and Biosystems, School of Chemical Engineering, Aalto University P.O. Box 16300 Espoo FI-00076 Finland orlando.rojas@ubc.ca marcobeaumont1@gmail.com; Department of Materials Engineering (DEMa), Federal University of São Carlos (UFSCar) Rod. Washington Luís km 235 São Carlos SP 13565-905 Brazil; Materials Chemistry Division, Department of Chemistry, University of Helsinki AI Virtasen aukio 1 FI-00560 Helsinki Finland; Surface Measurement Systems Ltd. Rosemont Rd Wembley London HA0 4PE UK; Departments of Chemical & Biological Engineering, 2360 East Mall; Chemistry, 2036 Main Mall, and Wood Science, 2424 Main Mall, The University of British Columbia Vancouver BC V6T 1Z3 Canada

## Abstract

A new regioselective route is introduced for surface modification of biological colloids in the presence of water. Taking the case of cellulose nanofibers (CNFs), we demonstrate a site-specific (93% selective) reaction between the primary surface hydroxyl groups (C6-OH) of cellulose and acyl imidazoles. CNFs bearing C6-acetyl and C6-isobutyryl groups, with a degree of substitution of up to 1 mmol g^−1^ are obtained upon surface esterification, affording CNFs of adjustable surface energy. The morphological and structural features of the nanofibers remain largely unaffected, but the regioselective surface reactions enable tailoring of their interfacial interactions, as demonstrated in oil/water Pickering emulsions. Our method precludes the need for drying or exchange with organic solvents for surface esterification, otherwise needed in the synthesis of esterified colloids and polysaccharides. Moreover, the method is well suited for application at high-solid content, opening the possibility for implementation in reactive extrusion and compounding. The proposed acylation is introduced as a sustainable approach that benefits from the presence of water and affords a high chemical substitution selectivity.

## Abstract 2

We report a versatile and simple preparation of hydrophobic cellulose nanofibers through a spatially confined modification of the primary surface hydroxyl groups.

## Graphical abstract

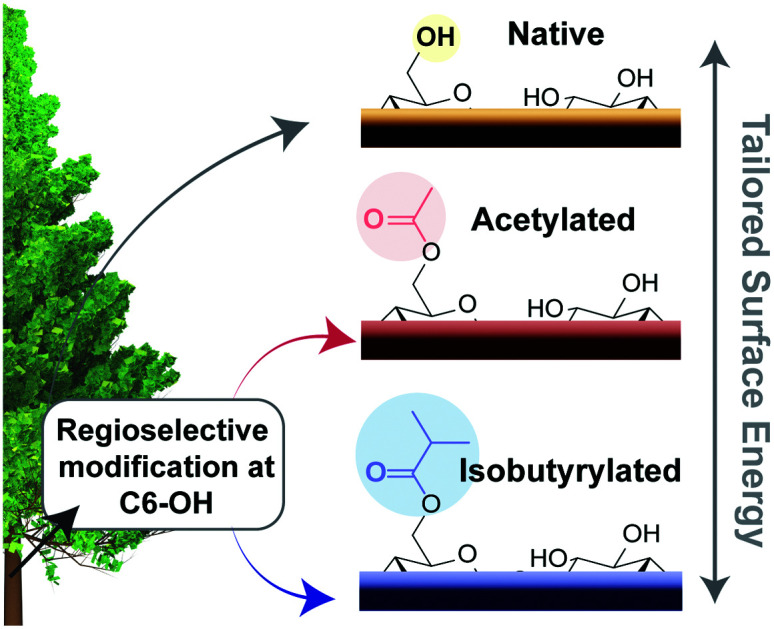

## Introduction

The run towards renewable nanomaterials is increasing in pace, and efforts are aimed at the isolation and modification of nanoparticles derived from structured biomass. Biological colloids, especially cellulose nanofibers (CNFs), are promising examples in this regard given the access to a wide variety of sources (*e.g.*, forestry and agricultural side-streams). In contrast to nanofibers from synthetic polymers, the cellulosic counterparts are obtained according to a top-down approach. The latter takes advantage of the hierarchical fibrous structure of plants with primary structural building blocks formed by the cellulose (nano)fibers. These entities offer unique intrinsic properties and can be assembled into materials^1^ with outstanding mechanical properties^2–4^ and specific surface area (SSA).^5,6^ The 2,2,6,6-tetramethylpiperidine-1-oxyl radical (TEMPO)-mediated oxidation has been introduced to install carboxylate groups onto cellulose in a regioselective manner.^7,8^ This reaction has enabled effortless fibrillation of fibers into individual TEMPO-oxidized CNFs. Unfortunately, several challenges still remain for a wide adoption of TEMPO oxidation, including costs, toxicity, and the chemical degradation that can occur during the oxidation, resulting in a functional cellulose with a significantly lower molar mass than the starting material.^9^ Such effects ultimately decrease the mechanical performance of cellulosic materials,^10^ setting a limit for TEMPO-mediated oxidation and, generally, a restriction of the introduced functionalities to hydrophilic moieties.

Chemically modified, hydrophobic CNFs are usually prepared by esterification to aliphatic esters preferably confined to the CNF surface. Related processes are in high demand given the need to endow CNFs with water resistance.^11,12^ So far, these modifications have been conducted either with dried cellulosic materials^12–14^ or in organic solvents, after solvent exchange.^11^ The required extensive water removal from cellulose in general leads to near irreversible formation of inter-fibrillar bonds, significant reduction of SSA and aspect ratio and reactivity – a phenomenon termed as “hornification”.^15,16^ This effect ultimately reduces dispersibility, water retention capacity,^15^ and the quality and performance of the obtained nanofibers in application.^17,18^ Moreover, full removal of water from cellulose is an enormous challenge, given the high hygroscopicity of cellulose and its strong interactions with water.^19^ As an alternative, solvent exchange of water-swollen fibers with an organic solvent avoids drying of cellulose but requires large solvent volumes and time- and energy consuming processing steps.^11^

While a plethora of chemical modification routes have been reported for (nano)celluloses, surface modifications are distinctive given that they do not affect the bulk of the material, *i.e.*, the crystallinity of the sample is preserved, and its molar mass is not reduced. This is not the case for the frequently used acid-catalyzed modifications with acetic anhydride in toluene/acetic acid to prepare acetylated nanocelluloses,^11,20^ as well as similar approaches that are based on the industrial heterogeneous acid-catalyzed acetylation.^21^ In addition, surface esterification of dried cellulose and subsequent fibrillation into CNFs requires considerable energy, *e.g.*, extensive ball-milling coupled with ultra-sonication.^12,14^ This also leads to a reduction in the cellulose molecular weight and crystallinity,^12,14^ also compromising its polymeric chain integrity.^22^

The surface-confined esterifications herein take place only at the accessible hydroxyl groups of the fibers, and do not influence the hierarchical structure of native cellulose and its crystalline domains. These form the basis of its inherently astonishing mechanical properties.^23,24^ Taking in consideration the challenges of surface modification, it is clear that the current methods to obtain, for example, surface-acetylated cellulose, are still far from meeting the need for a straightforward and sustainable process. In terms of sustainability, the main obstacles of the current methods relate to the high energy demand to completely remove water from cellulose, and the need for large volumes of organic solvents used in the solvent exchange of water. In addition, most esterification approaches are based on hazardous and non-sustainable solvents, mostly toluene,^25^ and require considerable energy input during the reaction, as heating^11,20^ or milling.^12,14^

**Fig. 1:**
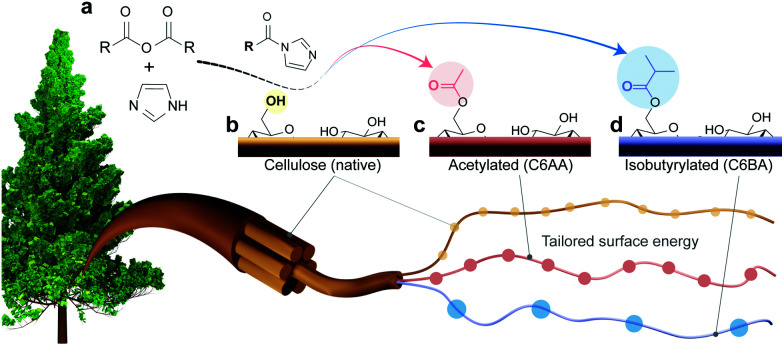
(a) C6-Regioselective esterification of cellulose in the presence of water. (b) Cellulose with imidazole-catalyzed reaction using (c) acetic or (d) isobutyric anhydrides to yield cellulose nanofibers with tailored surface energy.

We recently introduced a method for surface esterification of CNFs that tolerates water and occurs under mild conditions.^16^ This approach is based on the use of *N*-acetylimidazole as acetylation agent, which has also been shown to enable regioselective modification of dried cellulose fibers.^26^ However, this recent method lacks versatility since it only enables introduction of acetyl groups and it has not been demonstrated so far that it allows also the regioselective esterification of never-dried cellulose fibers, which is preferable to produce high-performance CNFs. Herein, we introduce a facile and versatile approach for the regioselective introduction of hydrophobic ester moieties onto the primary surface hydroxyl groups (C6-OH) of cellulose (Fig. 1a–d). The modification is sustainable and tackles most issues of current esterification methods, as no drying of cellulose or solvent-exchanges are necessary. In addition, the reaction is energy-efficient proceeding at room temperature; and uses an acetone/water mixture as solvent, which has less hazard and environmental impact than traditionally used organic solvents.^27^ We introduced successfully isobutyryl and acetyl moieties and, in an effort to fully describe the role of the substituent group, we extensively characterized the native and modified CNFs, for surface energy and interactions with water, highlighting the interfacial activity using model multiphase systems.

## Results and discussion

We introduce a method that enables direct esterification of never-dried cellulose through reaction of *N*-acylimidazoles, formed *in situ*, with the accessible hydroxyl groups at the surface of cellulose. This route is offered as an alternative to those that require either (a) removal of water from wet cellulose pulp, *e.g.* through spray/evaporative/freeze-drying, which can impair the mechanical performance of the produced nanocelluloses,^17,18^ or (b) solvent exchange, which in turn requires a high volume of organic solvents and centrifugation steps. The present approach expands our previous wet acetylation method that used a solution of *N*-acetylimidazole in DMSO.^16^ We replaced DMSO, an environmentally problematic solvent, with a greener alternative, acetone.^25^ This brought also the advantage during work-up, namely, the lower fiber swelling in acetone/water enabled more efficient washing for solvent removal as in case of prior DMSO/water system.^28^ Herein, most notably, and compared to previous efforts, we demonstrate that the wet esterification approach can be extended to different *N*-acylimidazoles, which are produced *in situ* through reaction of carboxylic acid anhydrides with imidazole (Fig. 1a).

**Fig. 2:**
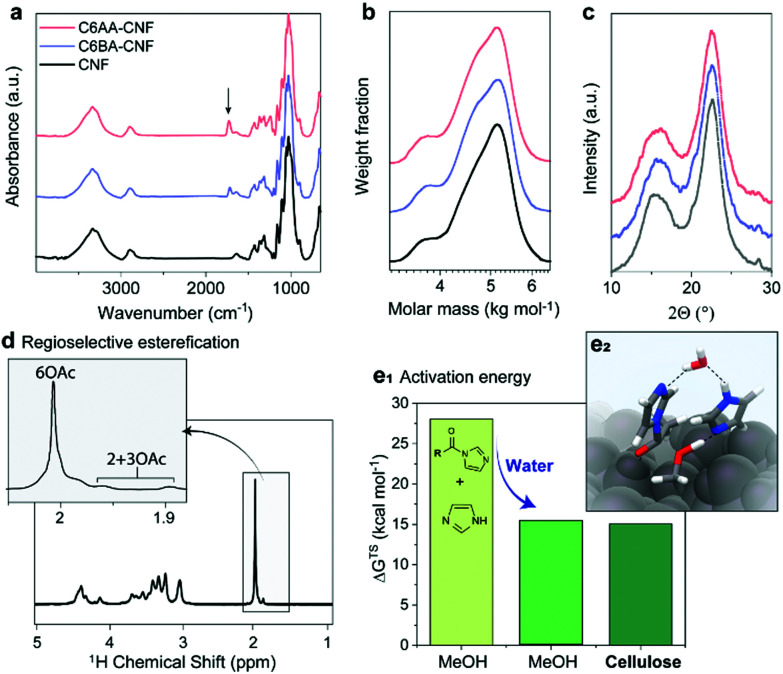
Chemical properties of acetylated (C6AA-CNF), isobutyrylated (C6BA-CNF), and native (CNF) cellulose nanofibers. The successful introduction of the ester groups is demonstrated through the presence of the IR carbonyl band (at 1727 cm^−1^) (a) and the diffusion-edited ^1^H-nuclear magnetic resonance spectrum (d). The procedure is mild and does not significantly impact the polymer chain integrity (b) and crystalline structure (c), as determined by gel permeation chromatography and wide-angle X-ray diffraction, respectively. The esterification is largely regioselective (d) and can be carried out in the presence of water (e1). In fact, the Gibb's free energy (Δ*G*^TS^) activation barrier was previously calculated for acetylation of methanol (MeOH) and cellulose with *N*-acetylimidazole and we show that water decreases the activation barrier and is further decreased through specific interactions with the cellulose surface (e2). Data for panels e1 and e2 were reproduced from the literature.^26^

Never-dried cellulose fibers (50 wt% solid content and 50 wt% water) were mixed with an imidazole solution in acetone before addition of either acetic anhydride or isobutyric anhydride to induce the formation of the respective *N*-acylimidazole. We chose the branched isobutyryl group to introduce higher hydrophobicity than the acetyl one, while exhibiting high colloidal stability in aqueous dispersions. The latter was not the case for longer alkyl chains, such as hexanoyl group (data not shown). The wet esterification proceeded spontaneously in the absence of heating to yield acetylated (C6AA) and isobutyrylated (C6BA) cellulose fibers, respectively. The modified fibers were further processed into CNFs through microfluidization. The infrared spectra in Fig. 2a indicate the successful introduction of the ester moieties, while other analyses (gel permeation chromatography and wide-angle X-ray diffraction) confirmed the success of the reaction under mild conditions (Fig. 2b and c). The crystalline structure of cellulose was not affected by the modification, while the weight-averaged degree of polymerization was only slightly reduced (Table 1). The latter is in contrast to regioselective modification *via* TEMPO-oxidation, which is known to cause significant polymer degradation, reducing the degree of polymerization by *ca.* 30–40%, even if performed under acidic or neutral conditions.^29,30^ As determined by nuclear magnetic resonance (NMR), the C6AA and C6BA samples featured degrees of substitution (DS) of 0.17 and 0.10, respectively, accounting to functionalization degrees between 1.0 and 0.6 mmol g^−1^. The regioselectivity of the esterification with *N*-acetylimidazole was confirmed by NMR (Fig. 2d), which showed that 93% of the C6-OH were modified (based on hydroxyl reactivity, the remaining 7% substitution mostly occurs at C2-OH). Usually, regioselective acetylation of cellulose requires the use of protecting groups and multi-step protocols.^31^ The high regioselectivity of acylation, with *in situ N*-acylimidazole, is rationalized by the decreased accessibility of the secondary hydroxyl groups, in presence of water.^26^

**Table 1:** Physical, mechanical, and surface properties of films formed with native and C6-esterified cellulose nanofibers

Feature	CNF	C6AA-CNF	C6BA-CNF
*γ*^d^^a^ (mJ m^−2^)	48.6	30.4	37.5
*γ*^ab^^a^ (mJ m^−2^)	23.3	7.9	16.1
*γ*^t^^a^ (mJ m^−2^)	71.8	38.8	54.0
*γ*^ab^/*γ*^t^^a^	0.32	0.20	0.30
*k*_acid_/*k*_base_	0.29	0.25	0.41
Specific surface area^c^ (m^2^ g^−1^)	158	168	164
Degree of polymerization^b^	969	895	899
Degree of crystallinity (%)	52	52	52
Tensile strength (MPa)	165 ± 20	186 ± 11	179 ± 12
Elastic modulus (GPa)	6.5 ± 0.3	7.5 ± 0.3	7.1 ± 0.3

a Median surface energies are reported: *γ*^d^, dispersive; *γ*^ab^, specific (acid–base); *γ*^t^, total surface energies and *γ*^ab^/*γ*^t^ ratio.

b Weight-averaged value.

c Determined by DVS (water vapor) according to the Brunauer–Emmett–Teller model.

Apart from enabling regioselective modification, the presence of water also promotes the reaction (Fig. S10†). The acetylation of never-dried (wet) fibers proceeds at significantly higher reaction rate if compared to the acetylation of dried fibers. This indicates a direct involvement of water in the reaction mechanism.^26^ As further confirmation, we calculated the Gibb's free energy of activation (gas-phase acetylation in methanol) and found that it was significantly reduced, by 56%, in the presence of water (Fig. 2e1 and e2). Additional modelling of the surface acetylation of the cellulose Iβ allomorph revealed a further decrease in activation energy due to interactions of *N*-acetylimidazole with the cellulose surface.

**Fig. 3:**
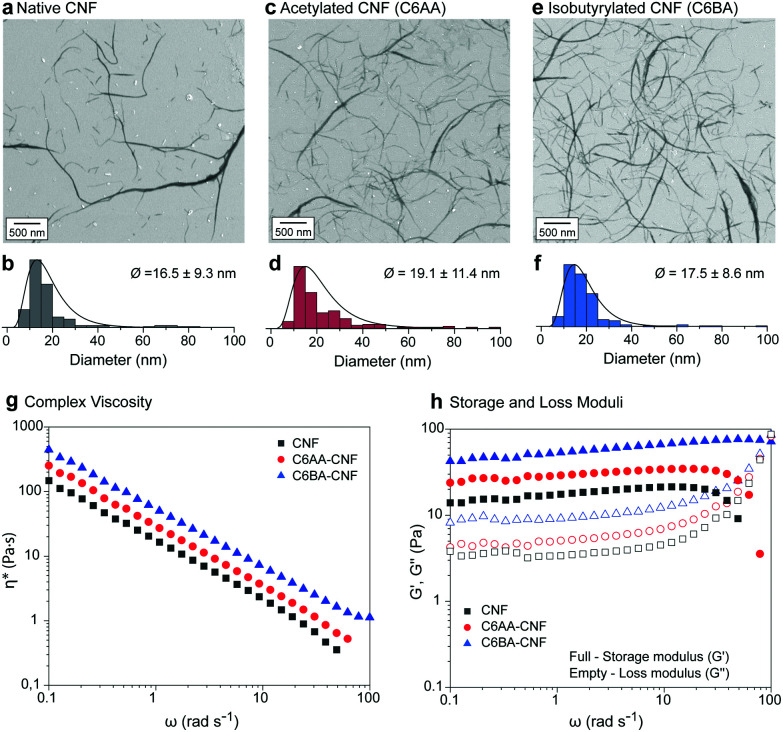
Negative contrast SEM images and diameter distribution (lognormal) of native CNF (a and b) and its esterified counterparts, namely C6AA-CNF (c and d) and C6BA-CNF (e and f). Oscillatory rheological profiles of aqueous suspensions (1.5 wt%) of CNF, C6AA-CNF, and C6BA-CNF: (g) flow profiles, and (h) storage and loss moduli.

Earlier reports showed that partial esterification of cellulose in the presence of protic organic solvents increased the defibrillation tendency.^11,13,16^ In our solvent system (acetone/water) this effect was not significant due to the lower swelling of the cellulose structure, which restricts the modification to its surface, also maintaining the integrity of the isolated colloids. The CNFs were *ca.* 20 nm in diameter (Fig. 3a–f) and their length spanned the range of several hundreds of nanometers (>600 nm) to few microns (*ca.* 5 μm). Note: The diameters of the nanofibers were obtained from negative contrast SEM images following the procedures described by Mattos–Tardy *et al.*^32^ Nanofibers of this size are especially attractive to induce cohesion in particle composites.^3^ Tailoring CNF surface energy, for example, to achieve lower hydrophilicity, is expected to widen the use of CNFs as adhesives, and in multiphase materials. Overall, given the nanofiber dimensions, our regioselective modification enables a systematic evaluation of the effect of interfacial interactions of cellulose.

The morphological similarities of native CNF, C6AA-CNF, and C6BA-CNF were further confirmed by studying their rheological behavior (Fig. 3g and h). All nanofibers underwent pronounced shear thinning with apparent and complex viscosity profiles within the same range of values (Fig. S5†). However, the C6-OH-esterified CNF displayed slightly higher viscosity than their native analogues. Moreover, the complex viscosity indicated a dominant viscoelastic behavior for all nanofiber suspensions, with the yield stress point (*G*′ = *G*′′) shifting to higher shear stress values in the order C6BA-CNF > C6AA-CNF > CNF. The results cannot be explained by the nanofiber dimensions alone but their distinct self-interactions as well as interactions with water, both of which led to a ‘viscosifying’ effect. In parallel, C6AA-CNF and C6BA-CNF displayed remarkably higher water exclusion feature (Fig. S3a†) when compared native CNF, also favoring fiber-fiber interactions centered on hydrophobic effects. The formation of an excluded water shell around nanocelluloses is an additional factor explaining the high viscosity of the C6BA-CNF and C6AA-CNF suspensions, as shown even at low mass fraction.^33^

Compared to C6AA-CNF and C6BA-CNF, a higher water retention, and therefore higher swelling, was observed for native CNF (Fig. S3a†). C6AA-CNF and C6BA-CNF, in turn, retained water (Fig. S3a†), but we speculate that the higher molecular steric hindrance at their nanofiber–nanofiber interface created steric exclusion in the nanofiber network and consequently increasing water excluded volume and leading to higher viscosity. Altogether, our proposed C6-regioselective hydrophobization can be taken as a toolbox for fine tuning the colloidal behavior of CNF, for instance to manipulate associative interactions (Fig. S3b†) and the rheological behavior (Fig. 3g and h), both of which are central to several technological efforts (*e.g.*, filament spinning^34^ and 3D printing^35^). We demonstrate the effect of C6-esterfication over the colloidal behavior of CNFs by its induced precipitation in the presence of various fractions of ethanol at increase *G*-forces. We show that C6-esterfied CNFs are more stable in conditions that would normally coagulate native CNF (Fig. S4†).

**Fig. 4:**
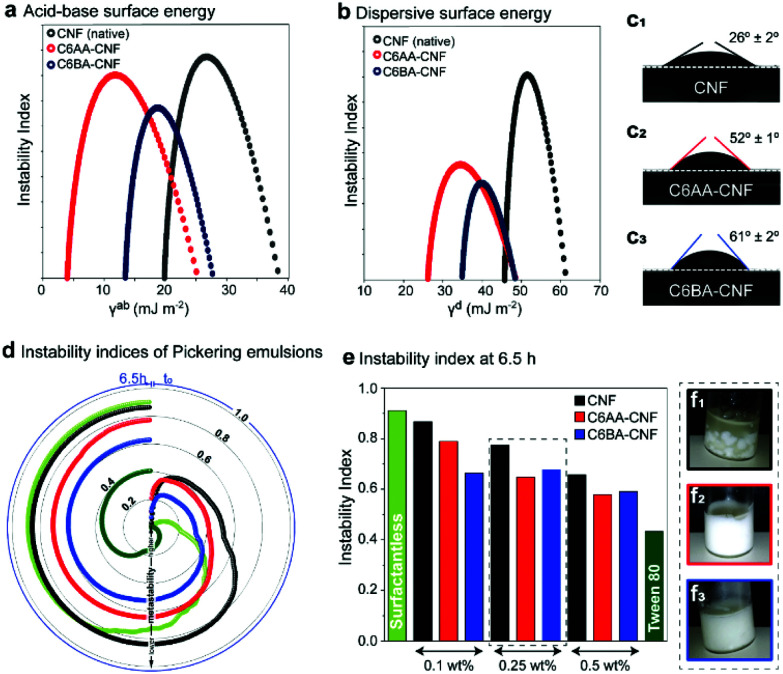
The acid–base and dispersive surface energy of unmodified cellulose nanofibers (CNF) is shifted to lower values upon modification due to the introduction of alkyl ester groups (C6AA-CNF in red and C6BA-CNF in blue) (a and b). Due to the higher surface coverage this effect is more pronounced for C6AA-CNF. The water contact angle depends on the alkyl chain length (c1–c3) and it is significantly higher for the isobutyrylated sample. This agrees with the instability indices of the Pickering emulsions prepared with sunflower oil (d and e), and the visual appearance of the respective Pickering emulsions from native CNF (f1), C6AA-CNF (f2), and C6BA-CNF (f3).

C6-OH-esterification allows for the tailoring of CNF for uses in multiphase systems. This is achieved by adjusting the surface energy and, thus, interfacial behavior at the air, liquid, and solid interfaces. As such, we used inverse gas chromatography (iGC) to elucidate the interactions of the unmodified CNF and the esterified CNFs with gases, namely acetone, acetonitrile, chloroform, dichloromethane, ethanol and ethyl acetate. The introduced ester groups reduced the total surface energy (*γ*^t^) of the CNF (Table 1). As expected, the specific (acid–base) term (*γ*^ab^) corresponding to polar, short-range interactions (mostly hydrogen bonds)^36,37^ was reduced, as the primary hydroxyl groups – now esterified – would be otherwise involved in polar gas interactions (Table 1 and Fig. 4a). In comparison to native CNF with a *γ*^ab^ of *ca.* 23 mJ m^−2^, C6AA-CNF featured the lowest *γ*^ab^, *ca.* 8 mJ m^−2^, followed by C6BA-CNF, *ca.* 16 mJ m^−2^. The lower specific surface energy of the former is attributed to the higher surface coverage by acetyl groups, as indicated by the higher DS (0.17 amounting to a modification of 73% of the maximum theoretical C6-OH, calculated from the cellulose crystallite size).^16,38^ The bulkier isobutyryl group was introduced to 43% of the available C6-OH groups. In a similar vein, the dispersive term *γ*^d^, referring to nonpolar, long-range interactions,^39^ influenced more significantly the total surface energy of C6AA-CNF, suggesting that the degree of surface coverage by the substituent moiety was more critical than its size. The surface chemistry of the samples was assessed using the Gutmann acid (*k*_acid_) and base (*k*_base_) numbers. Consistent with the literature,^39,40^ the *k*_base_ for C6AA-CNF and C6BA-CNF was consistently higher than *k*_acid_, indicating that the samples possessed preferential electron-donating (basic) abilities. Dependent on changes in charge density and relative abundance of the functional group, *k*_acid_/*k*_base_ can be shifted to slightly lower values, in case of C6AA-CNF due to the negative charge density of the carbonyl group. In case of longer alkyl chains, the positive charge of the alkyl chains dominates increasing the electron-withdrawing interactions (Fig. S1†). The observations discussed above for modified CNF in gaseous aprotic solvents differed from that in water, the protic analogue, both as vapor and as a liquid. Firstly, the capacity of CNF to adsorb water vapor was retained given the abundant density of hydroxyls on C2 and C3 (plus residual unmodified C6-OH) – note the similar equilibrium moisture contents (EMC) among the samples in dynamic vapor sorption (DVS; Fig. S7a†). Several factors are known to play a role in water interactions: while on one hand the reduced occurrence of hydroxyl groups is expected to lessen the EMC in C6-OH-esterified samples, the bulkier, less polar substituents might sterically impair interparticle cohesiveness and open the otherwise hidden sorption sites, accommodating more water molecules.^41^ Along these lines, we note that films made from C6-OH-esterified CNF displayed a higher SSA (Table 1). Importantly, the difference in moisture content at a given relative humidity, between adsorption (MC_ad_) and desorption (MC_des_) cycles, shows the hysteresis typical of cellulose with MC_des_ > MC_ad_.^19^ The higher hysteresis of native CNF (Fig. S6b†) was expected and may reflect the interplay between the increased occurrence of hydroxyls taking part in the (ad/de)sorption process, the larger moisture-induced structural changes, and the more pronounced hygroexpansion, all of which increase the hydrophilicity.^42,43^ Related features are confirmed by using liquid water as a probe, for instance, by measuring the water contact angle (WCA) of the films (Fig. 4c1): the introduction of acetyl groups doubled the WCA of unmodified CNF (Fig. 4c2), and it was further increased in the case of C6BA-CNF films (Fig. 4c3). Although the surface hydrophilicity of the CNF film was markedly reduced upon esterification, as one would expect, the size of the substituent ester group also affects the behavior: the higher WCA of C6BA-CNF, which is less substituted compared to C6AA-CNF, is explained by the fact that BA has more carbon atoms and is a bulkier moiety. This relationship between alkyl chain length and hydrophobization has been demonstrated with CNF butyrate and palmitate esters.^44^ The progressive variation of WCA with time gives further insight into the hydrophilic/hydrophobic character:^45^ during the first minute, the WCA of native CNF varied by *ca.* 17% while that of C6-OH-esterified CNF was more consistent, *ca.* 4% (Fig. S6†), pointing to the more limited influence of capillarity and polarity in the latter system.

By changing the surface energy of CNF, it is possible to tune the behavior at the oil/water interface, for instance in particle-stabilized colloidal multiphase liquid systems, the so-called Pickering emulsions. While the surface activities of all particles were all negligible, as demonstrated by the surface tension values of aqueous CNF suspensions (71 ± 1 mN m^−1^, determined by pendant drop in air – this value was constant during 30 min for all CNFs), the interfacial tension between a drop of water with suspended nanofibers and sunflower oil was reduced from the original value of 24 ± 1 mN m^−1^ by *ca.* 15% (CNF), 22% (C6AA-CNF), and 20% (C6BA-CNF). The small but clear reduction in interfacial tension of C6-OH-esterified CNF points to a better performance as a Pickering stabilizer. The macroscopic phase separation behavior within a centrifugal field was monitored *via* light transmittance (Fig. S8†). These profiles were used in the calculation of the emulsion instability index (EI), ranging from 0 to 1 and with lower values indicating higher kinetic stability. The evolution of the EI with time is depicted in Fig. 4d and e. As expected, O/W systems in the absence of any stabilizer presented the highest EI over the whole range. The emulsions stabilized with a nonionic surfactant, Tween 80, gave the lowest EI (Fig. 4e). The emulsions stabilized by C6-OH-esterification gave an EI that was lower than the other systems. After equilibration, the EI of all formulations corroborated the higher Pickering stabilization capacity of the C6-OH-esterified particles, which showed a higher tendency to adsorb at the interface given the presence of the hydrophobic moieties (Fig. 4f1–3).

Robust films were assembled from all the CNFs. The tensile strength and elastic moduli were higher for the C6-esterified CNF samples (Table 1 and Fig. S9†). It has been shown that the mechanical performance of cellulosic constructs can be improved by mild acetylation, *i.e.* without affecting their molar mass, and a subsequent fibrillation.^46,47^ Since fiber acetylation has been also shown to diminish the mechanical properties,^48^ the results of these nanofibrillated systems could be reasonably assumed to result from a more extensive fibrillation in the case of surface acetylated fibers. However, since our protocol did not significantly influence the dimensions of the prepared CNF, the increased mechanical properties can only be explained to originate from the regioselective introduction of functional groups. We speculate that the presence of alternating hydrophobic functional groups enables the formation of stronger cohesive interactions upon consolidation of the respective nanofiber suspension.

The SSA of the materials displayed similar values for all CNFs, confirming a similar nanofibrillar assembly for all of them. Entanglement and interconnectivity of the nanonetwork plays a fundamental role in transferring cohesion from single nanofibers to their macroscaled constructs. Given the higher mechanical properties of the C6-esterifeid samples, one can infer that nanofiber–nanofiber interactions played a determining role to enable efficient stress transfer mechanisms across the material. Steric hindrance and interfibrillar repulsion are expected to take place in the C6-OH-esterified nanofibers, which could enable a better densification of the system, for example, by drying after consolidation. The effects of interfibrillar repulsion as far as the formation of a well-organized nanofiber network have been previously discussed in terms of electrostatic interactions (as applicable to TEMPO-oxidized CNF);^49^ however, the present case of esterified CNF opens further opportunities for CNF deployment.

## Conclusions

We report for the first time a versatile and simple preparation of esterified CNF by using a highly regioselective modification (hydrophobization) of cellulose. Acetyl and isobutyryl units were successfully introduced (functionalization degree of 0.6 to 1.0 mmol g^−1^) following *in situ* formation of *N*-acetylimidazoles and *N*-isobutyrylimidazole from the corresponding anhydrides. The modification was confirmed to be selective to the surface of cellulose and to preserve the integrity of the crystalline structure and polymer chains. The regioselective introduction of acetyl and isobutyryl groups facilitated the production of nanofibers with tunable surface energy, as demonstrated by the measured interactions with aprotic gases, water vapor, and oil and water. Our results indicated that the regioselective modification increased the metastability of Pickering emulsions and enabled specific interactions with various fluids, depending on the surface coverage and size of the ester alkyl group. The increased suspension viscosity and storage moduli as well as the mechanical properties of films produced from the nanofibers indicated the prominent role of cohesive inter-chain interactions. In this regard, the regioselective modification of CNF offers an opportunity beyond the state-of-the-art. Overall, we introduce a chemical pathway for the controlled hydrophobization of nanocelluloses in the presence of water. The introduced method allows installing a wide range of functionalities and expands the application prospects of cellulose-based colloids.

## Experimental

### Materials

Cellulose fibers of high purity (93% cellulose, 4% hemicellulose) were provided as never-dried bleached beech sulfite dissolving pulp (50 wt% solid content) by Lenzing AG (Lenzing, Austria) and used in the production of the esterified pulp samples (C6AA-Cell and C6BA-Cell). Acetic anhydride (≥99%), isobutyric anhydride (97%), imidazole (ACS reagent, ≥99%), and acetone (DMSO, ≥99%) were purchased from Sigma-Aldrich (Merck Life Science OY, Finland). The never-dried pulp was pretreated before modification with a coffee blender to increase its SSA. Sunflower seed oil from *Helianthus* (CAS no. 8001-21-6) was used as provided by Sigma-Aldrich (Saint Louis, MO, USA) to fabricate emulsions.

### Preparation of esterified cellulose nanofibers

The pre-treated never-dried cellulose fibers (5 g dry cellulose content, 50 wt% solid content, 31 mmol) were added into 21.5 mL of 3 M imidazole solution in acetone and mixed with a glass rod for 5 min. After 30 min of equilibration in a closed container, 31 mL of 1 M carboxylic acid anhydride* in acetone (* either with acetic anhydride to yield C6AA-Cell or isobutyric anhydride to yield C6BA-Cell) was added and the reaction mixture was stirred for 24 h at room temperature to complete the esterification. Afterwards, the suspensions were filtered to remove the solvent, washed twice with acetone (2 × 50 mL) and 1 M aqueous solution of NaHCO_3_ (2 × 50 mL) and then four times with DI water (4 × 50 mL). Successful removal of solid reactants by washing was confirmed by IR (Fig. S13†) and NMR was used to confirm complete removal of solvent residues on basis of reported NMR chemical shifts.^50^

The esterified fibers (quantitative mass yield) were diluted to 0.5 wt%, mixed in a kitchen blender and fibrillated by six passes in a microfluidizer (200 and 100 μm chambers at 2000 bar). The processed nanofibers, either acetylated (C6AA-CNF) or isobutyrylated (C6BA-CNF), were compared to native CNF, which was fibrillated at the same conditions.

### Structural and morphological characterization

#### Solution-state nuclear magnetic resonance (NMR) spectroscopy

To prepare the samples for NMR analysis, typically 50 mg of dried sample (C6AA- or C6BA-Cell) was added to a sealable sample vial and made up to 1 g by addition of stock [P_4444_][OAc] : DMSO-d_6_ (20 : 80 wt%) electrolyte solution.^51,52^ The samples were magnetically stirred at room temperature until they went clear, which typically took approx. 1 hour. If the samples did not go clear during that period, the temperature was increased to 60 °C. All NMR runs were recorded on a Bruker AVANCE NEO 600 MHz spectrometer equipped with a 5 mm SmartProbe™ set to 65 °C.

The diffusion-edited ^1^H experiment used a 1D bipolar-pulse pair with stimulated echo (BPPSTE)^53^ diffusion-ordered spectroscopy (DOSY) pulse sequence (Bruker pulse program ‘ledbpgp2s1d’), with 1 s relaxation delay (d1), 0.5 s acquisition time (aq), 16 dummy scans (ds), 128 transient scans (ns), a sweep-width (sw) of 20 ppm with the transmitter offset on 6.1 ppm (o1p), diffusion time (d20) of 200 ms, gradient recovery delay (d16) of 0.2 ms, eddy current delay (d21) of 5 ms, diffusion gradient pulse duration (p30) of 2.5 ms, and z-gradient strength (gpz6) of 90% at 50 G cm^−1^ (probe *z*-gradient strength). These conditions are specific to the NMR apparatus above and may need reoptimization for other systems. The diffusion-edited ^1^H spectra for the samples C6AA- and C6BA-cellulose fibers are shown in Fig. S11a and S12a.† The degree of substitution was determined from ^1^H-NMR diffusion-edited spectra of C6AA- and C6BA-Cell relating the protons from the glucose monomer unit to the protons of the acetyl or isobutyryl group, respectively. The acetyl peak in the spectrum of C6BA-Cell (Fig. S12a†) is as impurity neglected for the calculation of the degree of substitution.

The HSQC experiments used a multiplicity-edited phase sensitive HSQC sequence with echo/antiecho-TPPI gradient selection (Bruker pulse program ‘hsqcedetgp’).^54^ The parameters were as follows: spectral widths were 13 ppm and 165 ppm, with transmitter offsets (o1p) of 6.18 and 75 ppm, for ^1^H and ^13^C dimensions, respectively. The time-domain size (td1) in the indirectly detected ^13^C-dimension (f1) was 512, corresponding to 256 t1-increments for the real spectrum. There were 16 dummy scans and 64 scans, an acquisition time of 0.065 s for f2 and a relaxation delay of 1.5 s. Spectral resolution was increased by zero-filling, by setting the size of the spectra in both dimensions to 1024 Hz. Sine squared (90°) window functions were used in f1 and f2. Respective HSQC spectra are shown in Fig. S11b and S12b.†

The regioselectivity of the acetylation in presence of imidazole and acetic anhydride was determined by peak fitting of the ^1^H-NMR spectrum (Fig. S13†), according to a previously published work.^26^

#### Fourier-transform infrared (FTIR) spectroscopy

Attenuated total reflection (ATR) FTIR spectra were obtained using a Frontier IR single-range (PerkinElmer Inc., MA, USA) spectrometer equipped with a ZnSe ATR crystal and a LiTaO_3_ detector. Four scans per measurement were performed for each sample at a resolution of 4 cm^−1^. The spectra were base line corrected and normalized to one in the software Spectragryph (Dr Friedrich Menges, Germany).

#### Nanofiber morphology

Negative-contrast scanning electron microscopy (NegC SEM)^32^ was used to measure the widths and approximate length of the unmodified and modified CNFs. The dimensions were taken from 200 nanofibrils selected randomly from several images. To warrant an unbiased fiber selection, we drew diagonal lines across the NegC SEM images and measured all the fibers that crossed by them. High-resolution images were acquired in a field emission gum electron microscope (FEG-SEM; Zeiss Sigma VP, Germany) using the in-lens detector. Acceleration voltage and working distance were 1.5 kV and 6 mm, respectively.

#### Size-exclusion chromatography (SEC)

SEC was carried out on a Dionex Ultimate 3000 system equipped with four identical PLgel Mixed-A columns (Agilent, USA) and a Shodex RI-101 refractive index (RI) detector (Showa Denko K.K., Japan). The never-dried samples were dissolved upon successive solvent exchange steps in acetone, DMAc, and DMAc/LiCl (90 g L^−1^) before being diluted with DMAc for a final LiCl concentration of 9.0 g L^−1^, the eluent of the columns. The RI signal was calibrated against a series of 11 narrow pullulan standards (Standard Polymer Service and Fluka, Germany), with molecular weights ranging from 342 Da to 2560 kDa.

#### Wide-angle X-ray scattering (WAXS)

Native CNF, C6AA-CNF, and C6BA-CNF suspensions in water were cast onto polystyrene Petri dishes and allowed to dry into films at room temperature. Once properly dried and equilibrated at a low relative humidity atmosphere, the same-thickness samples were sandwiched between a thin Mylar sheet and a zero-diffraction background Si disk. The WAXS diffractograms were recorded on an X'Pert Alpha-1 (PANalytical B. V.) diffractometer using reflectance geometry at Bragg angles varying from 0 to 70°, with a step size of 0.001° and 1 s per step. The device operated with Cu Kα radiation, selected by a monochromator, at a voltage of 45 kV and a current of 40 mA. The sample holder was continuously rotated during the acquisition. Sample-free Mylar/Si disk was run at the same conditions and subtracted as a blank. The precursor cellulose pulp was analyzed likewise for comparison purposes.

### Surface energy and activity

#### Inverse gas chromatography (iGC)

All analyses were carried out on an iGC Surface Energy Analyzer (Surface Measurement Systems Ltd, UK). The data were analyzed using the advanced SEA Analysis Software.

The samples (about 5 mg) were packed into individual 4 mm ID silanized glass columns and run at a series of surface coverage with alkanes and polar probe molecules in order to determine the dispersive surface energy (*γ*^D^_s_) as well as the acid–base free energy of adsorption (Δ*G*_SP_). The sample column was pre-conditioned for 2 h at 30 °C and 0% relative humidity with 10 mL min^−1^ helium carrier gas. The experiment was conducted at 30 °C at a 10 mL min^−1^ total flow rate of helium and methane was used to determine the dead volume of the system.

The dispersive surface energy (*γ*^D^_s_) analysis was performed by measuring the net retention volume *V*_N_ (measured retention volume minus dead volume) for a series of *n*-alkane eluents (in this case: hexane, heptane, octane, and nonane). The Dorris and Gray method was applied during the analysis: a plot of *RT* ln(*V*_N_) *versus* the alkane carbon number at a certain surface coverage produces a linear correlation. The dispersive surface energy of the solid sample can be determined from the slope of the line according to the following equation:Slope = 2(*γ*_CH_2__*γ*^D^_s_)^½^ × *N*_A_*a*_CH_2__,where *γ*^D^_s_ is the dispersive component of the solid surface energy, *a*_CH_2__ is the cross-sectional area of a methylene group, *γ*_CH_2__ is the surface tension of a methylene group at the measurement temperature, and *N*_A_ is the Avogadro constant.

The specific contribution to the total surface energy (*γ*^AB^_s_) was obtained by first measuring the specific free energies of desorption for different polar probe molecules, Δ*G*_SP_. These values were determined by measuring the net retention volume of polar probe molecules (acetone, chloroform, ethyl acetate, toluene, and dichloromethane) on the samples. Polar probe points are located above the alkane straight line on the *RT* ln(*V*_N_) *versus P*_D_ plot. The vertical distance to the straight line is equal to the specific component of the free energy of desorption, Δ*G*_SP_. From the Δ*G*_SP_ values based on the van Oss approach, the specific surface energy (*γ*^AB^_s_) of the samples were calculated. The specific contribution is subdivided into an acid *γ*_s_^+^ and a base *γ*_s_^−^ parameter of the surface energy. In this approach, the Della Volpe scale is employed, with a pair of mono-functional acidic and basic probe molecules (dichloromethane, *γ*_s_^+^ = 124.58 mJ m^−2^ and ethyl acetate, *γ*_s_^−^ = 475.67 mJ m^−2^). The surface chemistry of the samples was assessed using the Gutmann acid (*K*_A_) and base (*K*_B_) numbers, determined based on the Gutmann approach using the following polar probes: dichloromethane, ethyl acetate, acetone, and chloroform. The *K*_A_ and *K*_B_ values of the samples were calculated using the Δ*G*_SP_ values of polar probes at a particular surface coverage and listed in Table 1.

To represent the heterogeneity of the samples in a more illustrative manner, the surface energy distributions are obtained by a point-by-point integration of the surface energy profiles, resulting in plots of *γ*^D^_s_, *γ*^AB^_s_, and *γ*^T^_s_ surface energies *versus* percentage of surface (area increment), as shown in Fig. 4a and b. The medians *γ*^D,50^_s_, *γ*^AB,40^_s_, and *γ*^T,50^_s_ are defined as the surface energies at which half of the population lies below this value listed in Table 1. The ratio of the *γ*^AB^_s_ and *γ*^T^_s_ indicates the surface polarity and it is also listed in Table 1.

The total work of cohesion was determined according to geometric mean method using the surface energy components of the samples:*W*^total^_Cohesion_ = 2[(*g*^D^_s_ × *g*^D^_s_)^½^ + (*g*_s_^+^ × *g*_s_^−^)^½^ + (*g*_s_^−^ × *g*_s_^+^)^½^].

#### Interfacial activity

The pendant drop method was used to assess the interfacial activity of the CNFs. A 20 μL drop of 0.25 wt% CNF (either pristine or esterified) suspension was injected into a cuvette filled with sunflower oil. The shape of the pendant drop was monitored over 30 min on a goniometer (Biolin Scientific, Theta Flex) and subsidized interfacial tension calculations.

#### Wettability

Aqueous suspensions of native and esterified CNF at 0.5 wt% were drop-cast onto silicon wafers and allowed to dry at room conditions. The sessile drop method was used to calculate the apparent contact angle between 6 μL drops of ultrapure water and the films. A goniometer (Biolin Scientific, Theta Flex) was used to record the droplet shape and calculate the contact angle every 1 s during 60 s.

### Emulsion formation and stability

#### Emulsification

Aqueous suspensions of native or esterified CNF at 0.1–0.5 wt% were mixed with sunflower oil at a volume ratio of 1 : 1. Water and sunflower oil were also mixed at the same ratio, but in the absence of CNF or any surface-active molecules (surfactantless; herein taken as negative control) or added with 0.25 wt% Tween 80 (positive control). The systems were tip-sonicated (500 W at 20% amplitude) for 3 min (7 s on/3 s off steps) in an ice bath to avoid excessive heating.

#### Metastability

The level of kinetic stability of the emulsions was evaluated in an accelerated fashion by centrifuging at 4000 rpm on a LUMiSizer dispersion analyzer (LUM GmbH, Germany). Emulsions (400 μL) were poured into 2 mm-optical pathway polycarbonate cuvettes, through which the transmittance of near-infrared (*λ* = 865 nm) radiation was monitored as the samples were horizontally centrifuged for 6.5 h at 25 °C. The transmittance profile throughout the cuvette led to the instability index, dimensionless quantity that ranges from 0 (higher kinetic stability) to 1 (higher kinetic instability).

## Conflicts of interest

There are no conflicts to declare.

## Supplementary Material

GC-023-D1GC02292J-s001
