# Supplementary material for: Regioselective and water-assisted surface esterification of never-dried cellulose: nanofibers with adjustable surface energy
Source: Green Chem. 2021 Aug 2;23(18):6966–74. doi: 10.1039/d1gc02292j (PMC8452180; doi:10.1039/d1gc02292j)
Supplement: GC-023-D1GC02292J-s001 [file GC-023-D1GC02292J-s001.pdf]

Electronic Supplementary Material (ESI) for Green Chemistry.
This journal is © The Royal Society of Chemistry 2021

Supplementary information (ESI)

Regioselective and water-assisted surface esterification of never-dried cellulose: nanofibers with adjustable surface energy

Marco Beaumont[a,b,#,], Caio G. Otoni[c,#], Bruno D. Mattos[b,#], Tetyana V. Koso[d], Roozbeh Abidnejad[b], Bin Zhao[b], Anett Kondor[e], Alistair W. T. King[d], Orlando J. Rojas[b,f,*]*

a. *Department of Chemistry, Institute of Chemistry for Renewable Resources, University of Natural Resources and Life Sciences Vienna (BOKU), Konrad-Lorenz-Straße 24, A-3430 Tulln, Austria.*
b. *Department of Bioproducts and Biosystems, School of Chemical Engineering, Aalto University, P.O. Box 16300, Espoo FI-00076, Finland.*
c. *Department of Materials Engineering (DEMa), Federal University of São Carlos (UFSCar), Rod. Washington Luís, km 235, São Carlos, SP 13565-905, Brazil.*
d. *Materials Chemistry Division, Department of Chemistry, University of Helsinki, AI Virtasen aukio 1, FI-00560 Helsinki, Finland.*
e. *Surface Measurement Systems Ltd., Rosemont Rd, Wembley, London HA0 4PE, UK.*
f. *Departments of Chemical & Biological Engineering, 2360 East Mall; Chemistry, 2036 Main Mall, and Wood Science, 2424 Main Mall, The University of British Columbia, Vancouver, BC V6T 1Z3, Canada.*

[#]These authors contributed equally to this work. *Corresponding authors: orlando.rojas@ubc.ca, marcobeaumont1@gmail.com

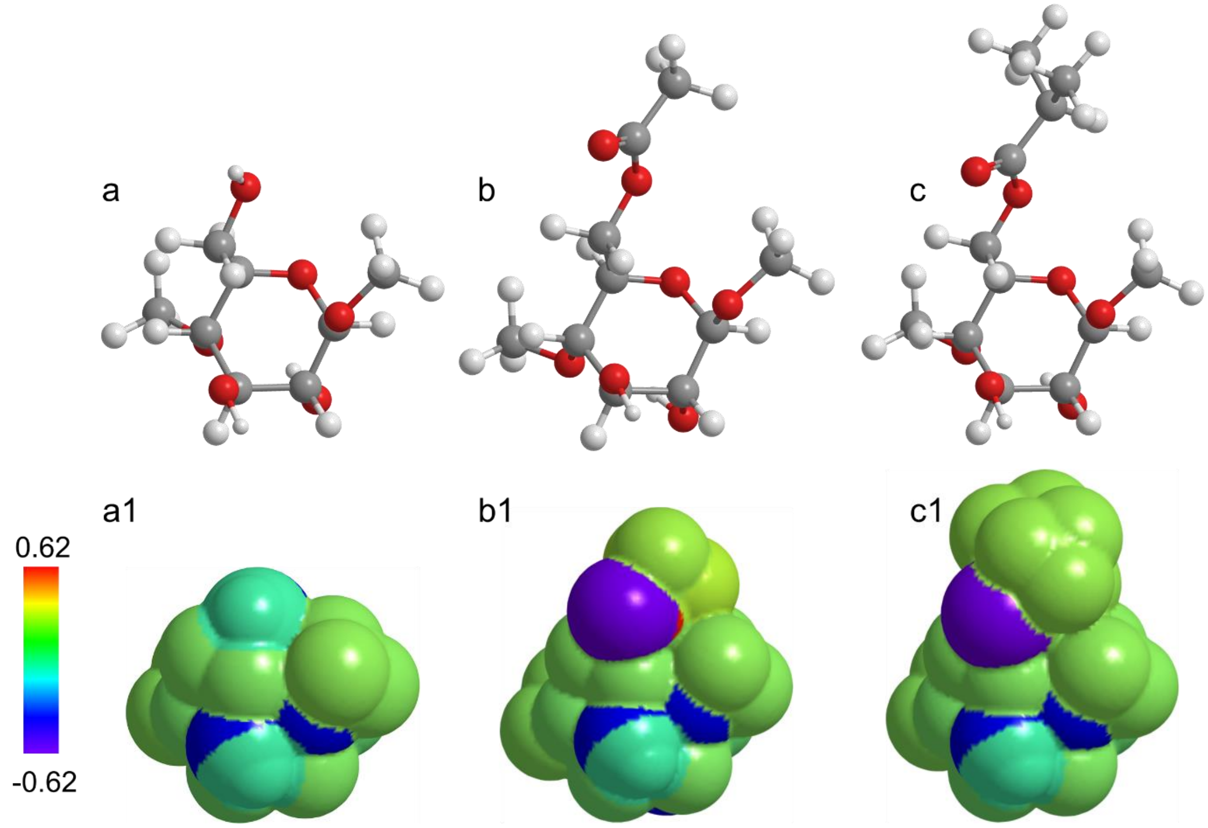

Figure S1. Molecular structures of 6-OH (a), 6-O-acetyl (b) and 6-O-*iso*-buturyl (c) 1,4-O,O'-dimethylglucopyranoside and visualized Hückel charge density of the corresponding units (a1-c1).

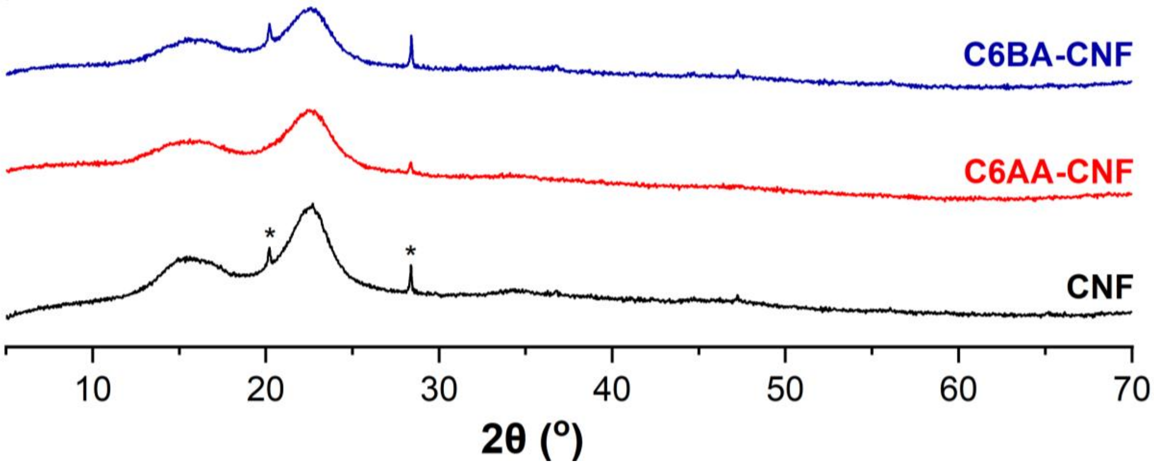

Figure S2. X-ray diffraction patterns of cellulose nanofiber films from native and C6-esterified cellulose nanofibers. Y-axis: intensity (a.u.). The asterisks highlight peaks assigned to the sample holder.

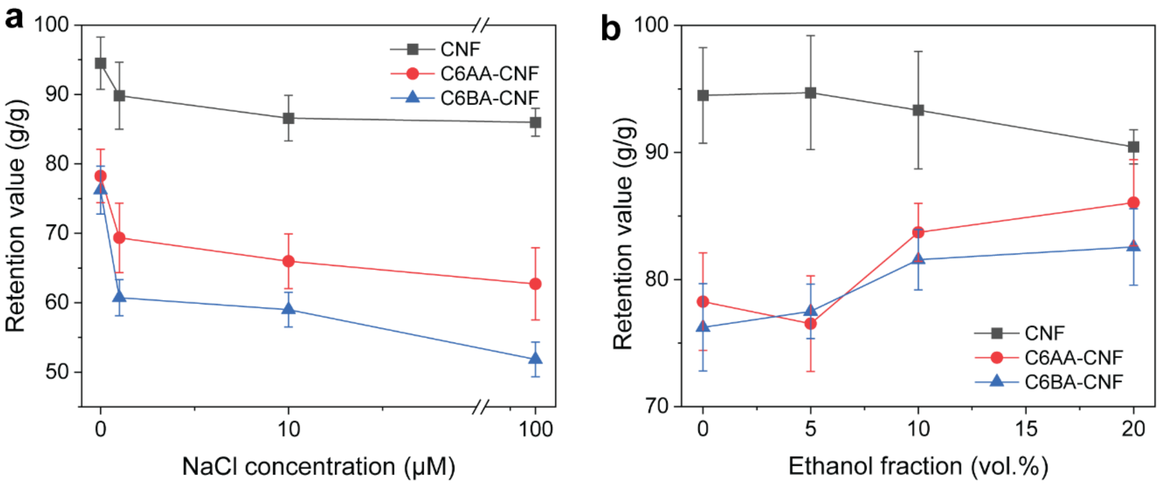

Figure S3. Water retention values for native and c&-esterified CNFs as a function of salt (**a**) and ethanol (**b**) concentration.

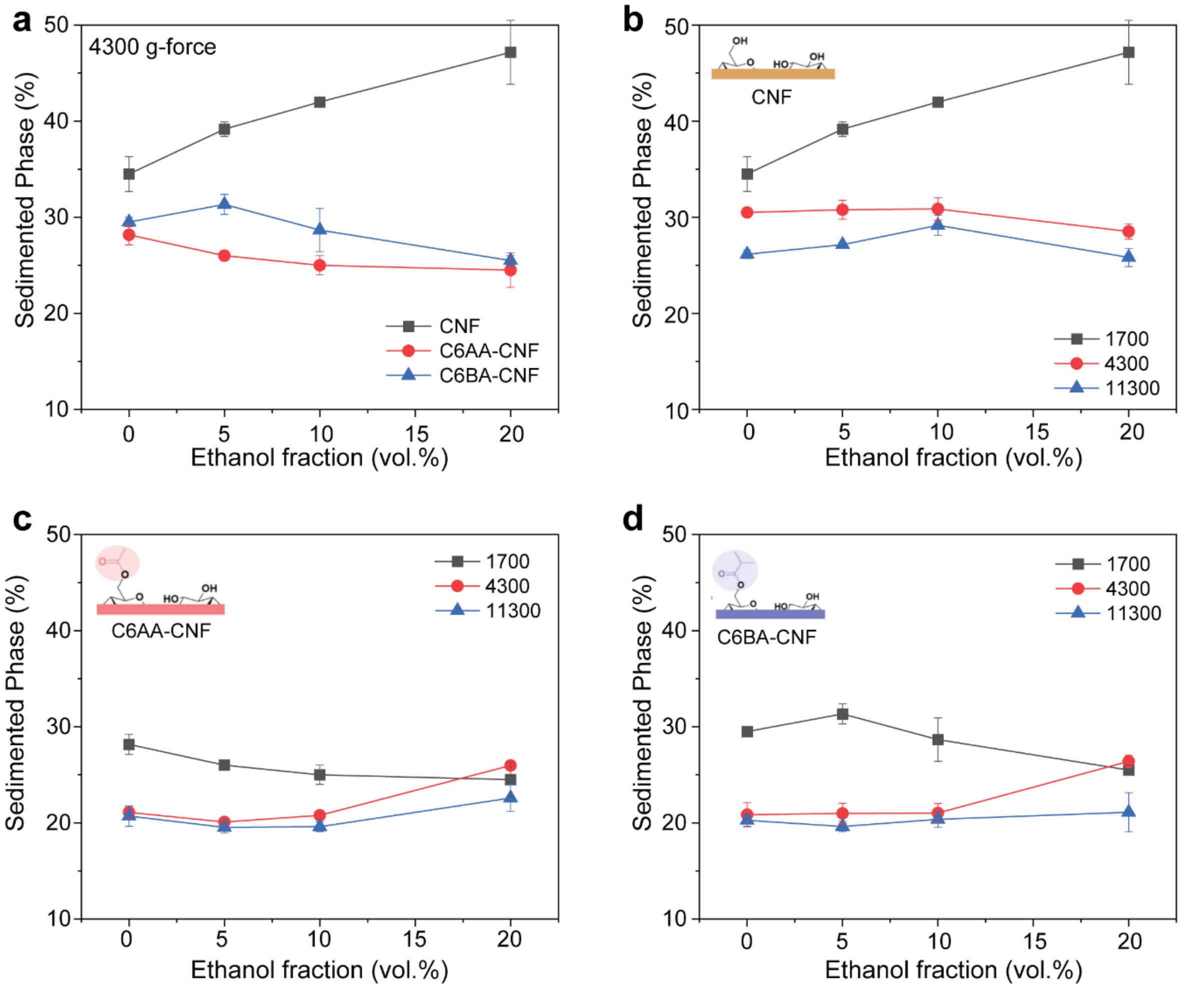

Figure S4. Colloidal stability (**a**) of native (**b**) and C6-esterfied CNFs (**c-d**) in suspensions containing given ethanol fractions exposed to increased G-forces. Note: ethanol is known to induce coagulation in cellulose.

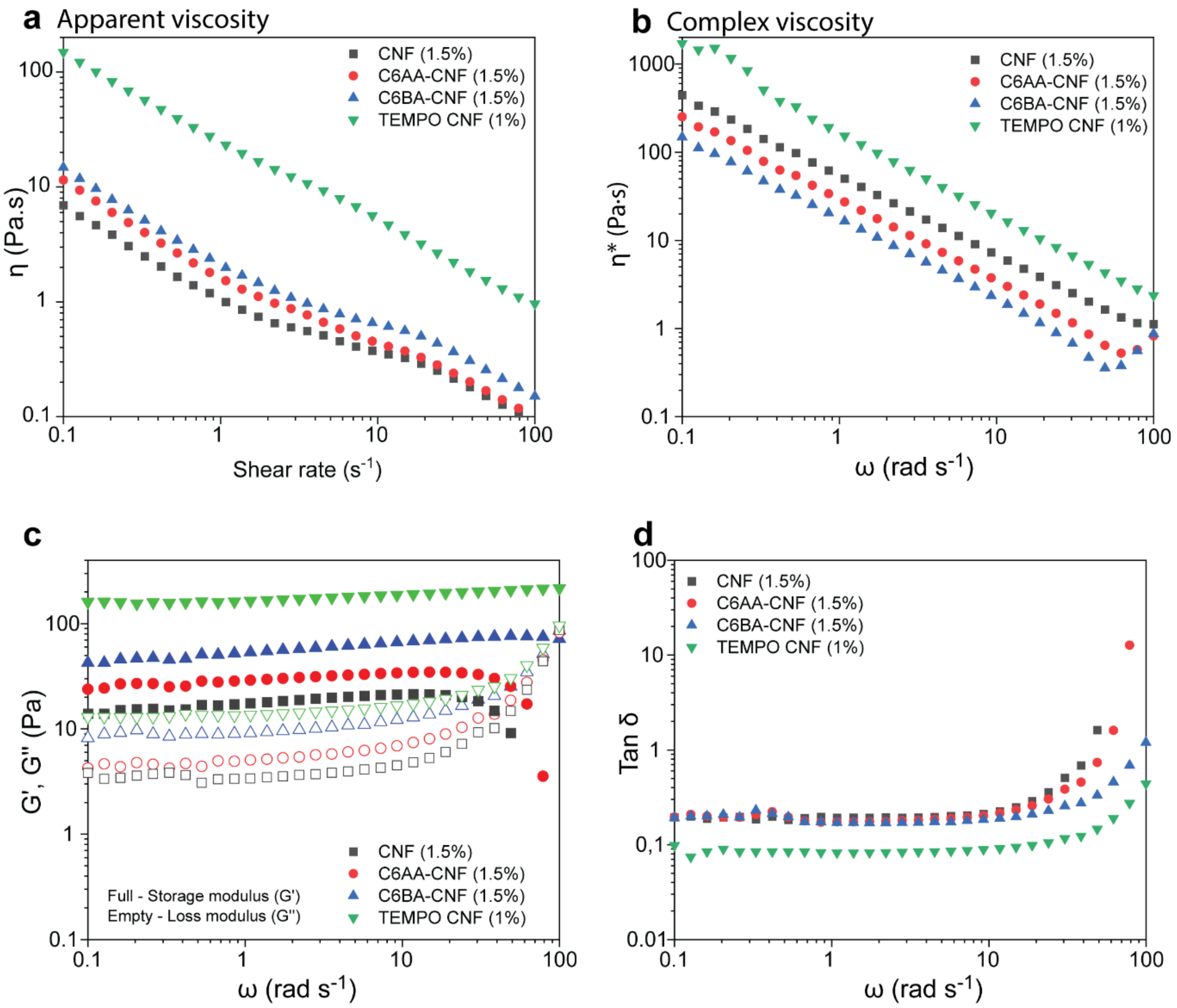

Figure S5. Apparent (**a**) and complex viscosity (**b-d**) for native CNF and C6-esterfified CNFs, as well as a comparison with TEMPO-CNF. Note that TEMPO-CNF was measure at lower mass fraction.

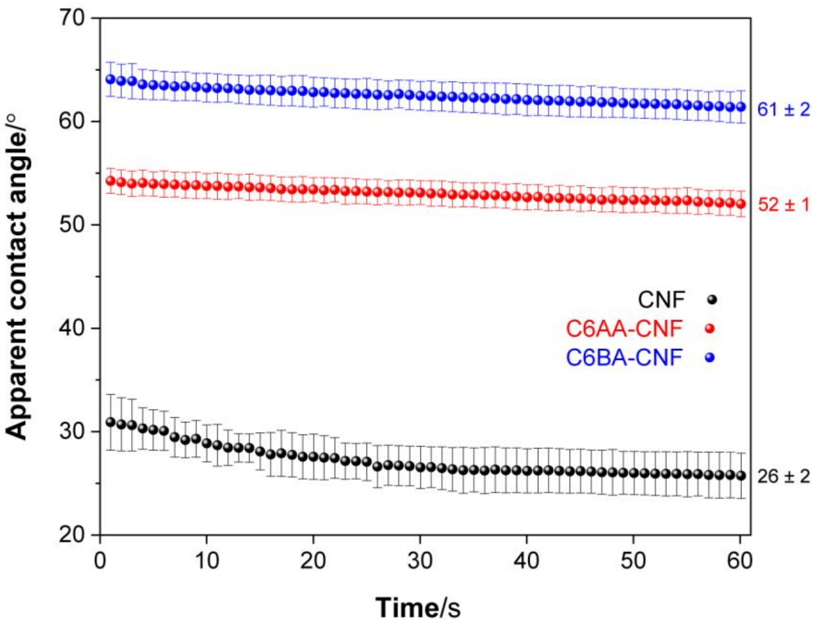

Figure S6. Evolution of the contact angle between films from native and C6-esterified cellulose nanofibers and liquid water.

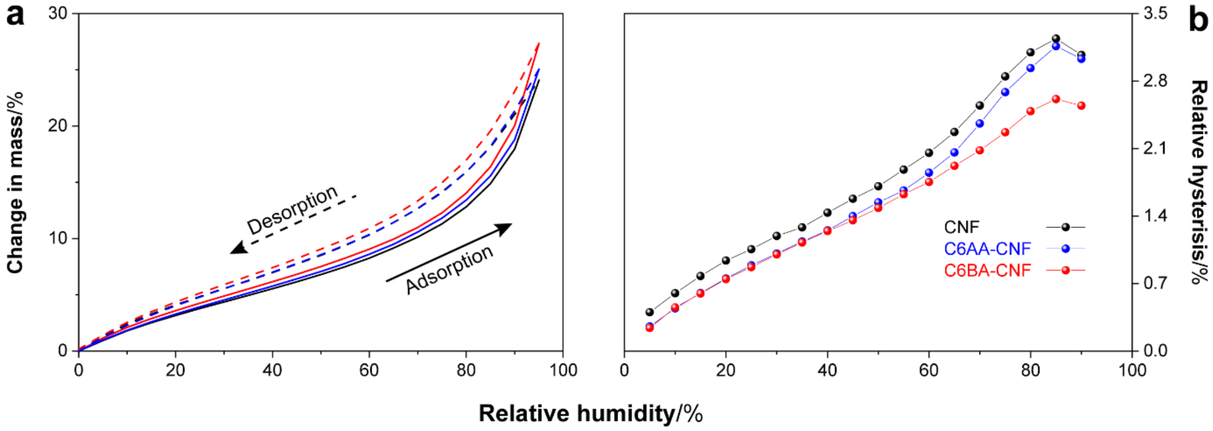

Figure S7. Dynamic vapor (ad/de)sorption **(a)** and hysteresis **(b)** profiles of films from native and C6-esterified cellulose nanofibers.

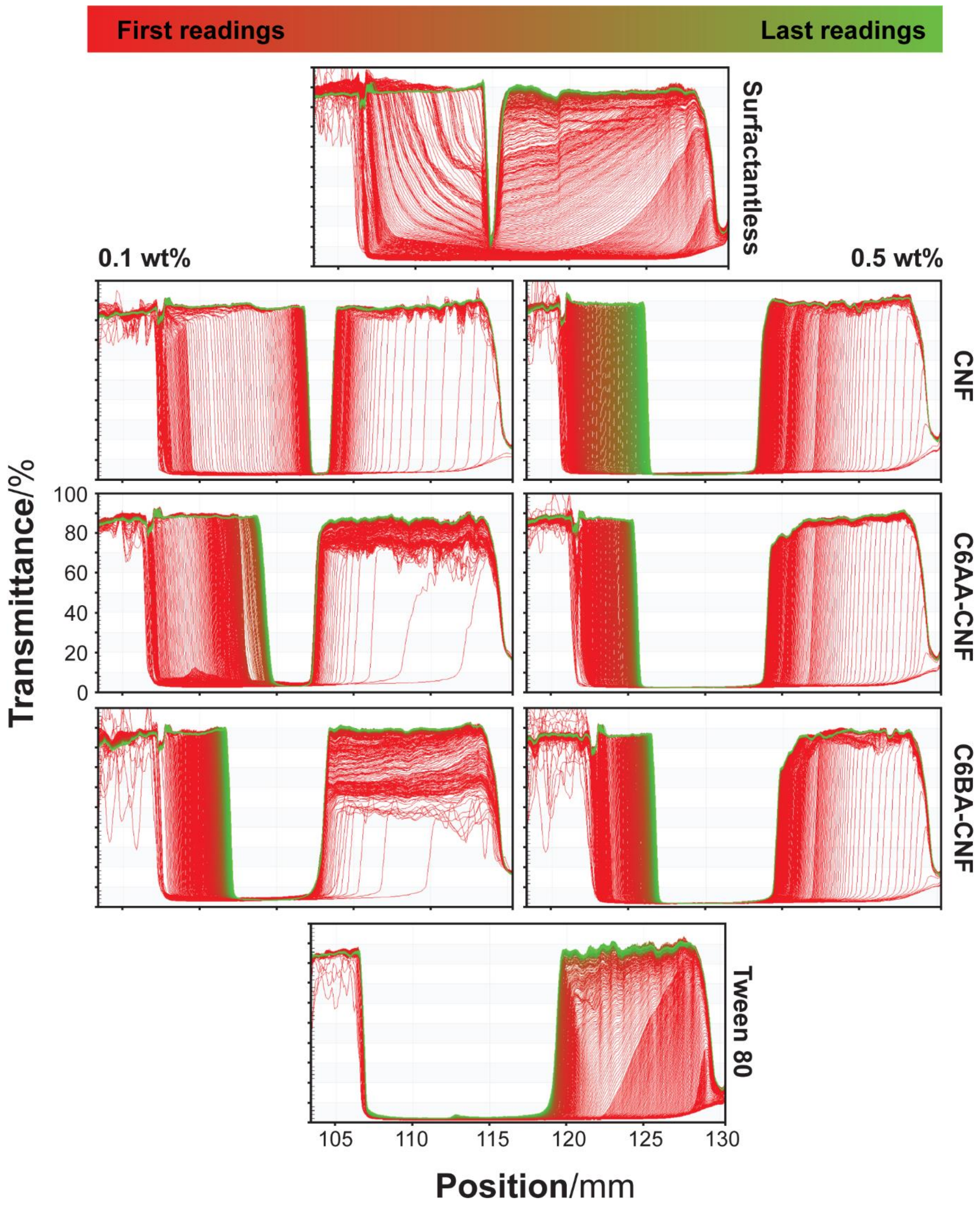

Figure S8. Typical transmission profiles through oil-in-water emulsions kinetically stabilized by native and C6-esterified cellulose nanofibers at 0.1 or 0.5 wt%. Controls are surfactantless (negative) and Tween 80 (positive). Profiles are resolved both in time (red to green, from older to newer acquisitions, respectively) and space (cuvette length).

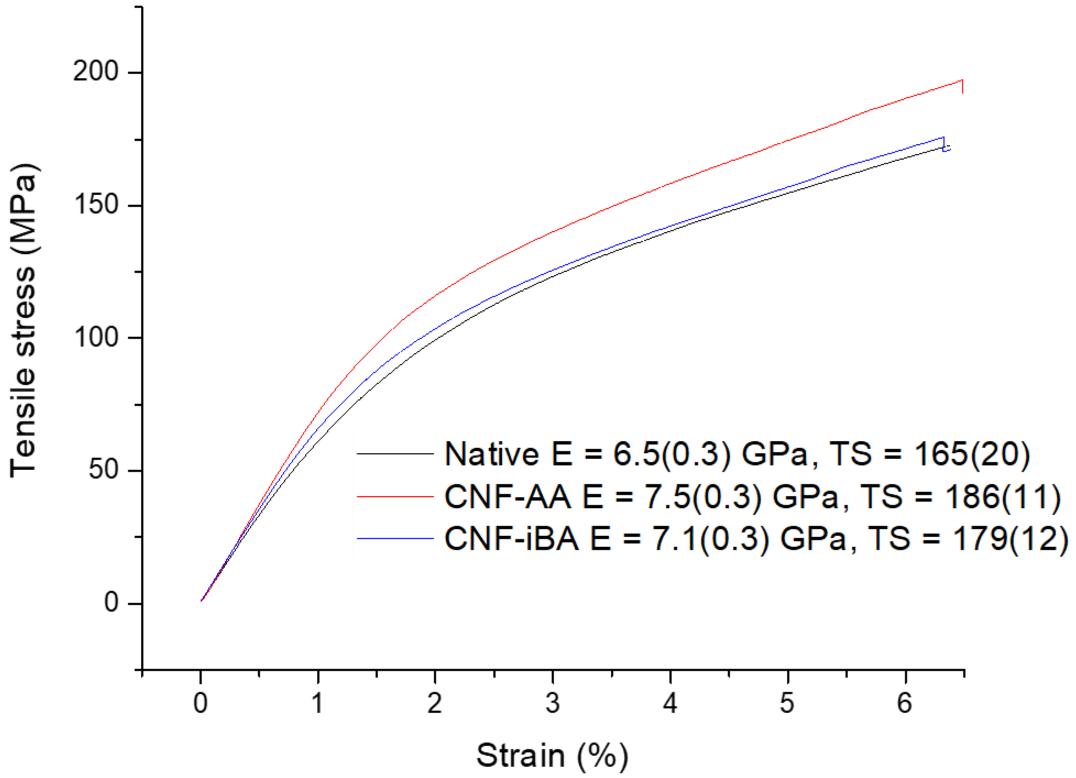

Figure S9. Typical tensile *versus* strain curves of films from native and C6-esterified cellulose nanofibers.

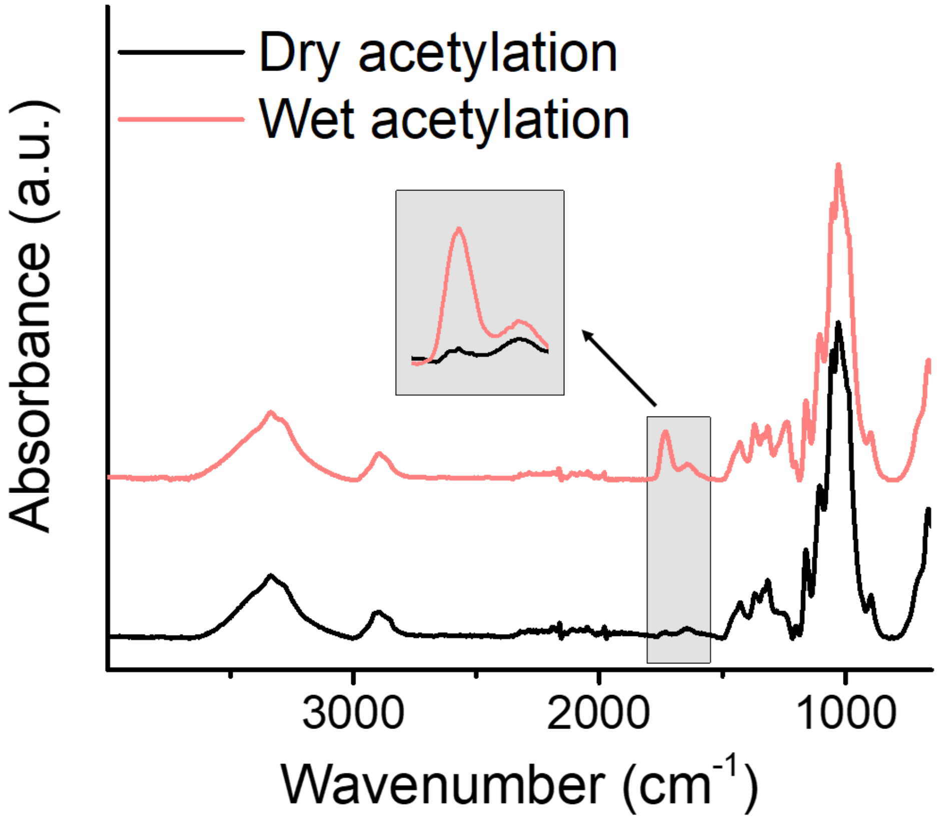

Figure S10. Effect of water on the reaction kinetics. Comparison of acetylation with acetic anhydride and imidazole of never-dried (wet) and dried (dry) cellulose fibers.

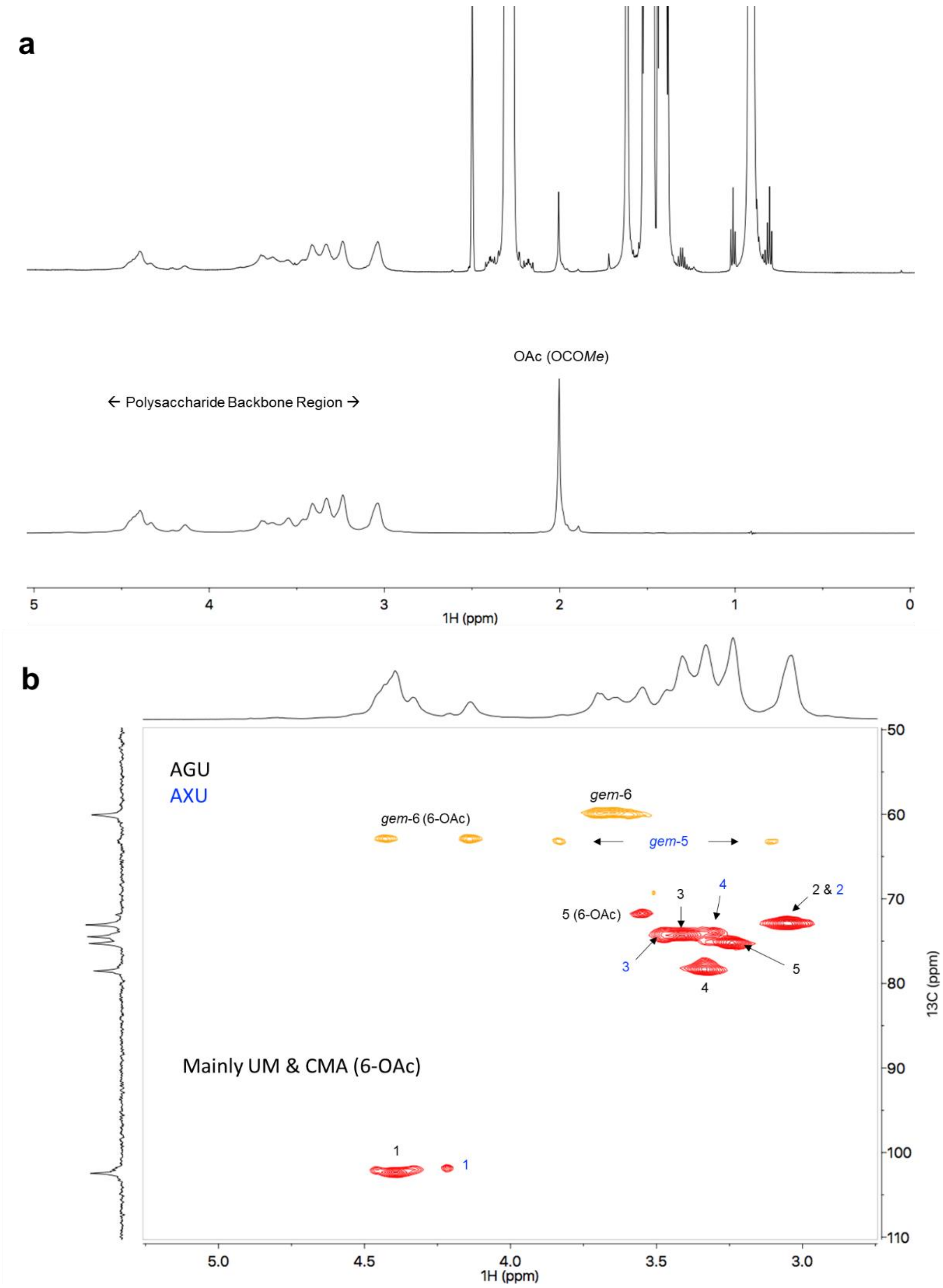

Figure S11. Diffusion-edited ^{1}H-NMR and multiplicity-edited HSQC of C6-OH-acetylated cellulose fibers.

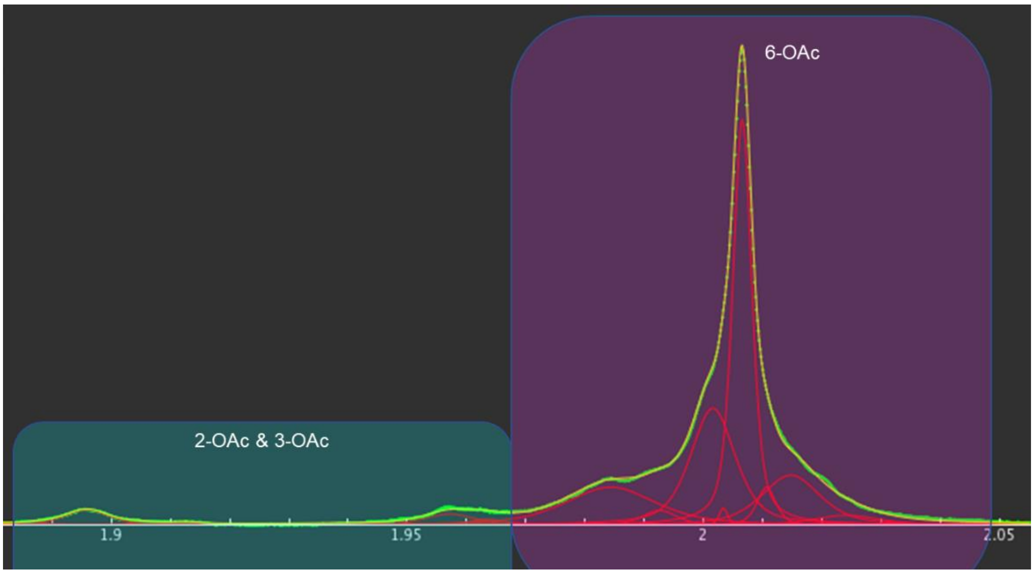

Figure S12. Peak fitting of the 6-OAc and 2+3-OAc peaks of acetylated cellulose fibers of the respective ^{1}H-NMR spectrum to determine the regioselectivity was conducted according to our recent work.[1]

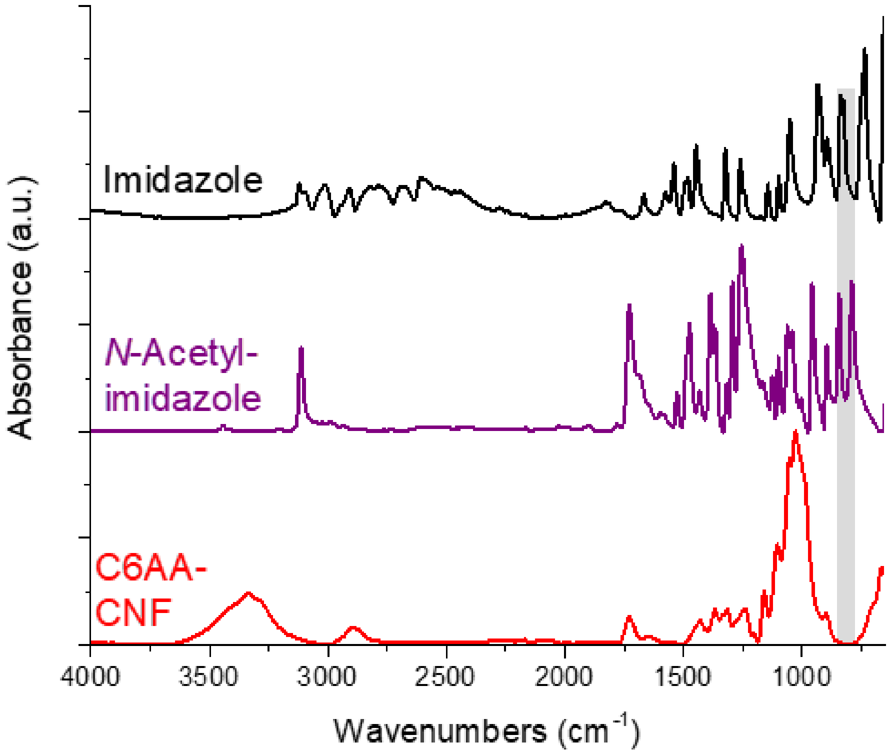

Figure S13. Infrared spectra of C6AA-CNF in comparison to used and formed reactants, imidazole and *N*-aetylimidazole, showing the complete removal of the latter ones during washing. The box highlights the region between 780 and 850 cm^{-1}.

Supplementary Experimental Section

Water retention value

Unmodified and modified CNFs suspension were diluted to 1% w/v and submitted to centrifugal forces to calculate their solvent retention capacity. Additionally, to pure water, three fractions of ethanol (5, 10, and 20 vol%) were added to the CNF suspensions in order to investigate the presence of cellulose coagulants on the retention values of the modified CNFs. Empty centrifuge tubes (15 mL) were dried at 103 ± 2 ºC to obtain the m_0. Then 3 mL of each suspension was added and centrifuged at 4000 *g* for 10 min. The supernatant was then removed, and the tubes were weighed to obtain m_{wet}. The tube containing the precipitated CNF was dried at 103 ± 2 ºC to obtain the m_{dry}. The water retention value (WRV, in g·g^{-1}) was calculated following the equation below.

$$WRV = \frac{m_{wet} - m_{dry}}{m_{dry} - m_0}$$

Dynamic vapor sorption (DVS)

Films from native and esterified CNFs, produced as described in the XRD methodology, were used as platforms for water vapor to adsorb/desorb upon changes in the environmental moisture conditions. The profiles were recorded gravimetrically on a DVS Intrinsic Plus analyzer (Surface Measurement Systems Ltd.). Film samples of ca. 15 mg were exposed to RH varying from 0% to 95% (adsorption cycle) and from 95% to 0% (desorption cycle), both at 5% steps that were finished when the sample weight varied less than 0.005%·min^{-1} within 10 min.

Rheology

The rheology of the native and C6-esterified CNFs was characterized on a MCR3000 rheometer (Anton Paar) equipped with a plate-plate geometry (steel, 25 mm diameter, 1.5 mm gap). All measurements were conducted at 23 ºC. Serrated plates were used to minimize wall slippage. The apparent viscosity (*h*) was measured as a function of the shear rate ($\dot{g}$) in the range between 0.1 and 100 s^{-1}. Amplitude sweep measurements of the storage (*G′*) and loss (*G″*) moduli were conducted at 1 Hz in the range of 0.1-100% to establish a strain amplitude (*g*) of 0.5% for the successive frequency sweep measurements. The angular frequency (*w*) of the oscillatory shear was varied between 0.1-100 rad·s^{-1} to obtain the corresponding *G′* and *G″*, as well as the complex viscosity (*h**).

Supplementary References

1 M. Beaumont, P. Jusner, N. Gierlinger, A. W. T. King, A. Potthast, O. J. Rojas and T. Rosenau, *Nat Commun*, 2021, **12**, 2513.
